# Supplementary material for: Establishment of chromatin accessibility by the conserved transcription factor Grainy head is developmentally regulated
Source: Development. 2020 Mar 11;147(5):dev185009. doi: 10.1242/dev.185009 (PMC10624965; doi:10.1242/dev.185009)
Supplement: Supplementary information [file develop-147-185009-s1.pdf]

Nevil Figure S1

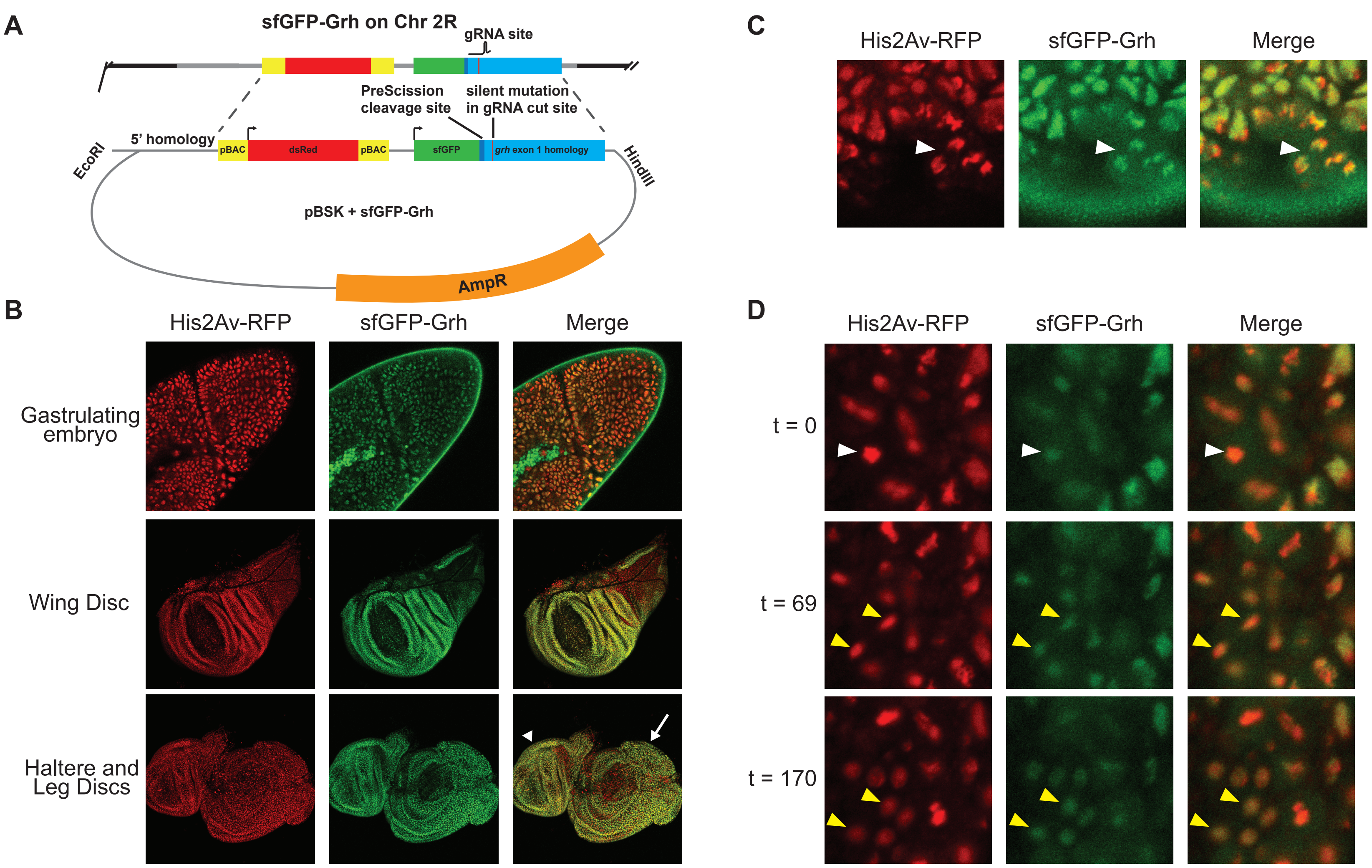

Figure S1. Endogenous tagging of Grh with sfGFP. (A) Scheme of sfGFP donor plasmid and predicted *grh* genomic locus after CRISPR/Cas9-mediated tagging. 1 kb homology arms (5' homology and exon 1 homology) flank a 3xP3-DsRed cassette inserted for screening. The DsRed cassette is flanked by piggyBac elements for precise excision after screening. sfGFP was inserted at the N-terminus of Grh, separated by a PreScission Protease cleavage site as a linker. (B) Confocal microscopy images of a *sfGFP-Grh; His2Av-RFP* embryo at gastrulation, larval wing disc, haltere disc (arrow head), and leg disc (arrow). (C) Confocoal microscopy image of *sfGFP-Grh; His2Av-RFP* embryo at gastrulation. White arrow indicates cell in anaphase. (D) Timecourse of confocoal microscopy images of *sfGFP-Grh; His2Av-RFP* embryo at gastrulation. White arrow indicates parent nucleus. Yellow arrows indicate daughter nuclei. t, time in seconds from initial image.

Development • Supplementary information

Nevil Figure S2

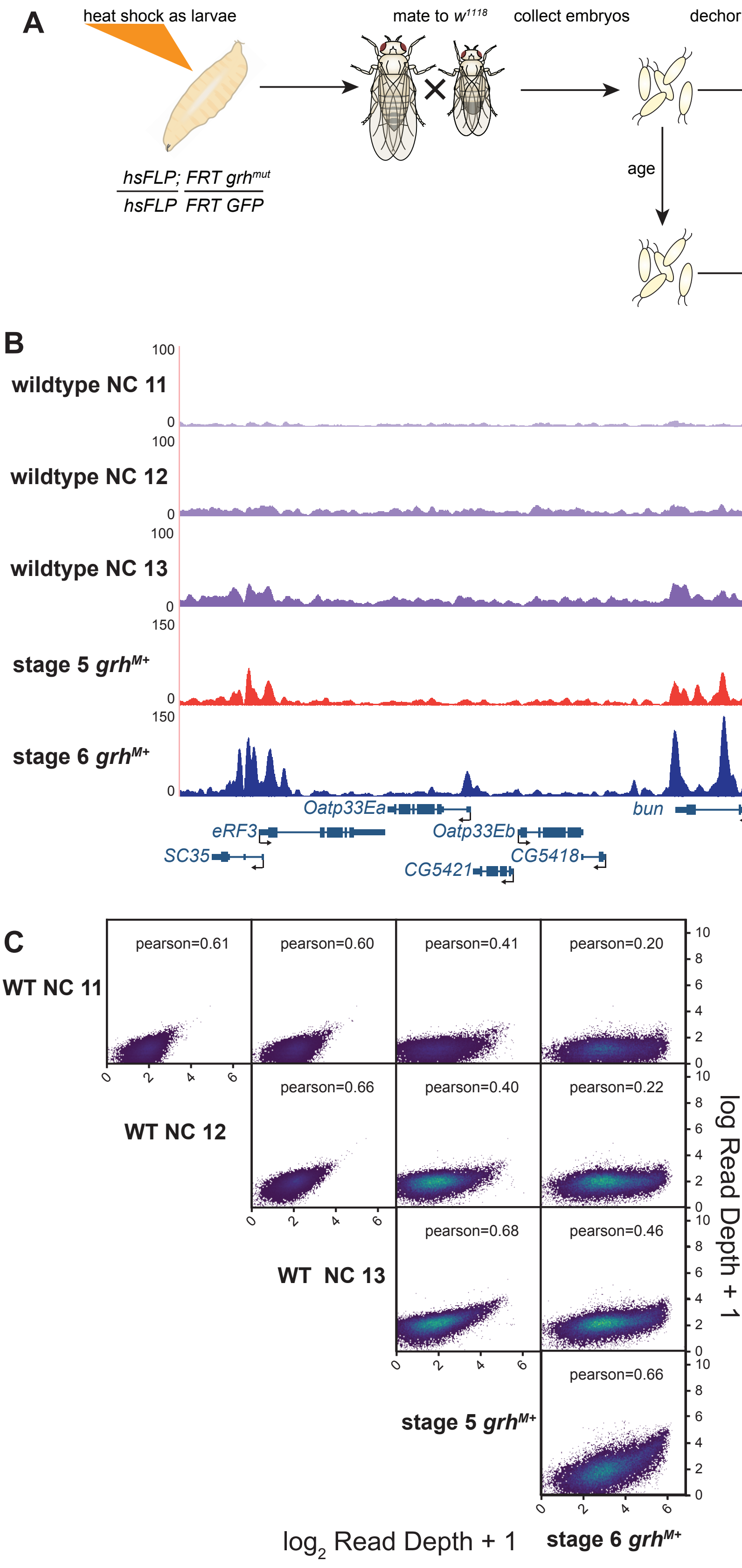

Figure S2. ATAC-seq on embryos depleted of maternally encoded Grh. (A) Scheme of heat shock FLP-FRT protocol used to generate germline mitotic clones lacking maternally encoded Grh. (B) UCSC genome browser track with published nuclear cycle (NC) 11, 12, and 13 ATAC-seq data from wild-type embryos (Blythe and Wieschaus, 2016) and wild-type stage 5 and stage 6 embryos. (C) Pearson correlation plots of published nuclear cycle (NC) 11, 12, and 13 ATAC-seq data from wild-type embryos (Blythe and Wieschaus, 2016) and wild-type stage 5 and stage 6 embryos at accessible regions of chromatin. Higher Pearson correlation coefficients indicate greater similarity between datasets. Data are transformed using $\log_2$ read depth + 1 for plotting, not for correlation calculation.

Development • Supplementary information

Nevil Figure S3

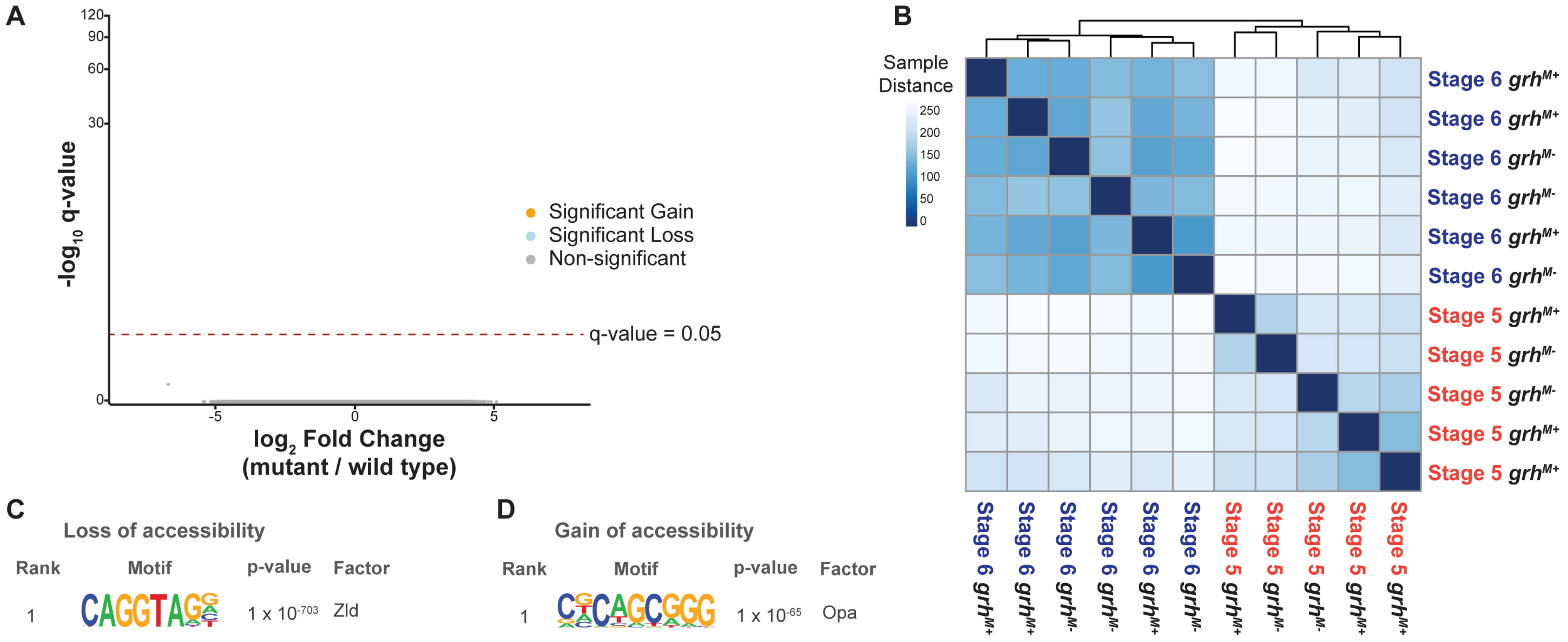

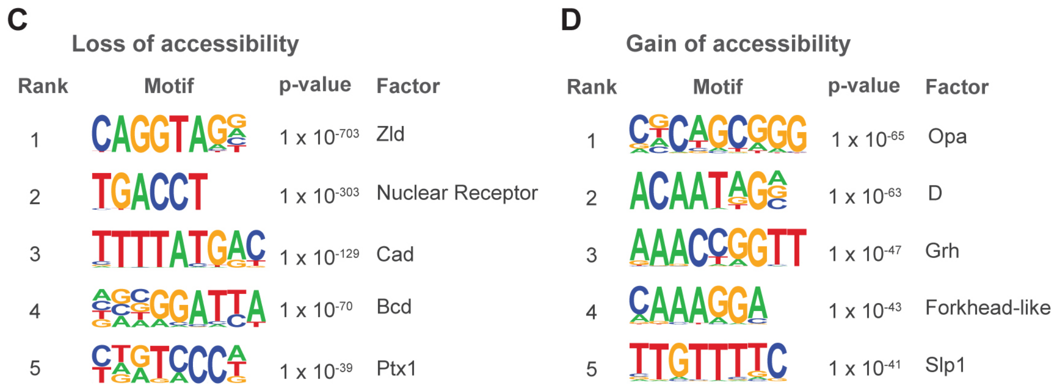

E

	Increased Accessibility	Decreased Accessibility	p-value
**Grh**	**13.9%**	4.5%	< 2.2 x 10^{-22}
**Zld**	22.5%	**33.8%**	< 2.2 x 10^{-22}

Figure S3. Stage is a better predictor of clustering than genotype. (A) Volcano plots of all accessible regions identified using the DESeq2 package in comparisons between stage 6 *grh* maternal depletion and heterozygous siblings (Love et al., 2014). Significance of change in accessibility reported by -$\log_{10}$ q-value on the y-axis, and magnitude of change by $\log_2$ fold change on the x-axis. Regions that significantly gain (orange) or lose (blue) accessibility are defined as those with a q-value < 0.05. Non-significant changes are those with a q-value > 0.05 (gray). (B) Hierarchical clustering of sample distances for single embryo ATAC-seq replicates of stage 5 and stage 6 maternal depletions (*grh*$^{M-}$) and sibling controls (*grh*$^{M+}$). Color and phylogenetic tree indicate sample-to-sample relatedness. (C) Top 5 motifs identified in *de novo* motif enrichment of regions that gain chromatin accessibility compared to all accessible regions at gastrulation. (D) Top 5 motifs identified in *de novo* motif enrichment of regions that lose chromatin accessibility compared to all accessible regions at gastrulation. (E) Percentages of ChIP-seq Grh (2-3hr AEL embryos) and Zld (cycle 14 embryos) peaks that overlap with sites that significantly increase or decrease in chromatin accessibility during gastrulation. p-values calculated using a two-sided Fisher's exact test comparing the enrichment of each transcription factor at differentially accessible sites.

Development • Supplementary information

Nevil Figure S4

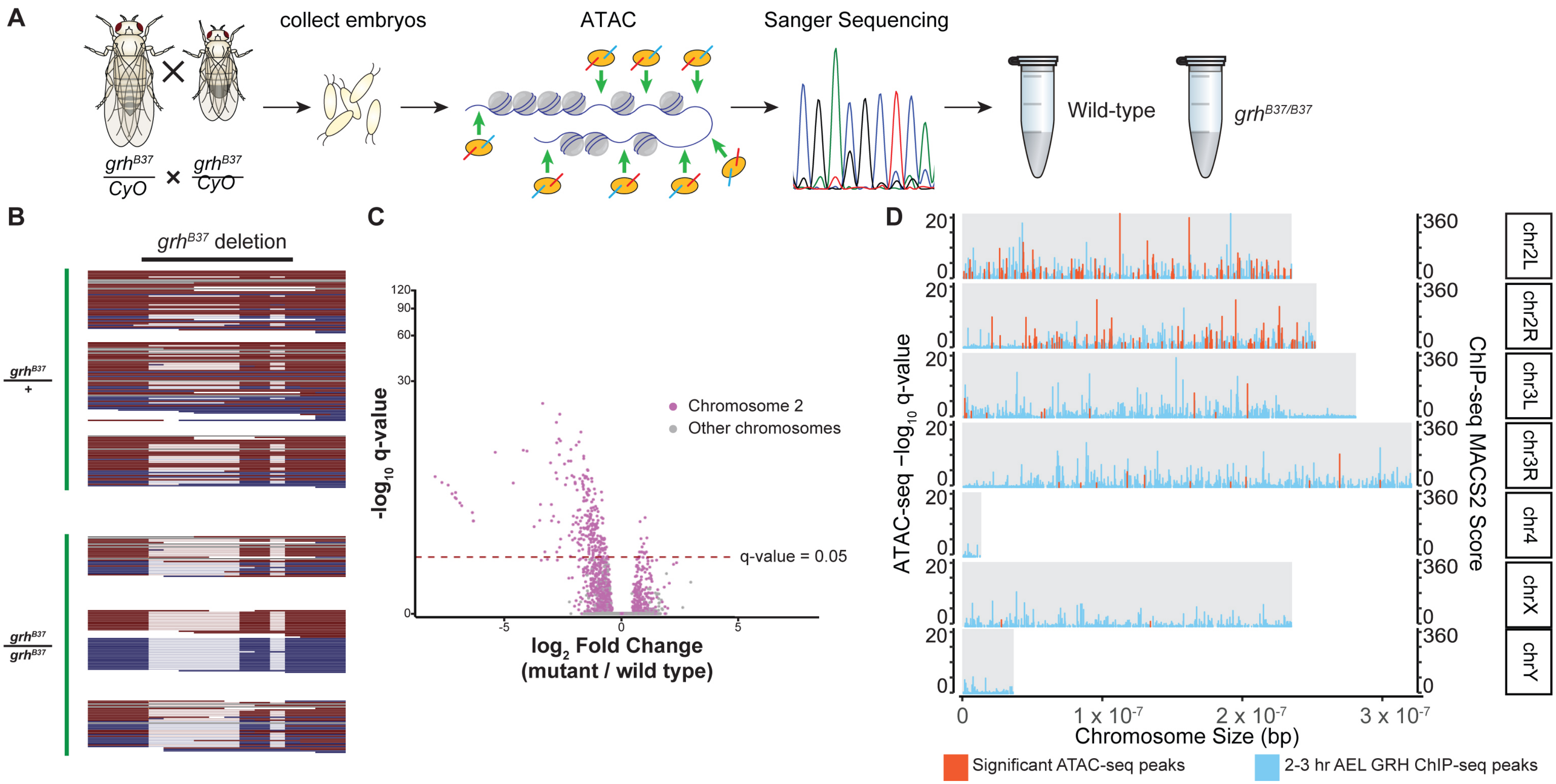

Figure S4. ATAC-seq on embryos depleted of zygotically expressed Grh. (A) Scheme used to collect and identify *grh*$^{B37/B37}$ embryos. Single embryos were collected for ATAC. After library preparation, the region spanning the *grh*^{B37} deletion was amplified by PCR and sequenced. Homozygous mutants were selected. (B) ATAC-seq reads aligned (red = reverse strand, blue = forward strand) to the wild type sequence that is deleted in the *grh*^{B37} allele. Each line is an individual read that spans the deletion, each block of reads represents a single replicate (three per genotype). Thick lines indicate overlap to the reference (wild type) sequence, whereas thin lines indicate lack of sequence overlap from the mutant allele. The lack of wild-type, spanning reads in the homozygous mutant indicates correct identification of mutants. (C) Volcano plot showing all accessible regions identified. Significance of change in accessibility reported by -log$_{10}$(q-value) on the y-axis, and magnitude of change by log$_2$(fold change) on the x-axis. Regions are colored by location on either chromosome 2 (purple) or on other chromosomes (gray). Data are identical to those in Figure 4B. (D) Location of significantly changing regions (red bars) of accessibility show the largest number and most significant (-log$_{10}$ q-value) are located on chromosome 2, the chromosome where the *grh*^{B37} allele has been maintained over a balancer allowing mutations to accrue and limiting mapping of sequencing reads. Plotting the location of Grh ChIP-seq peaks from Nevil et al. 2017 (blue bars, height by MACS2 score column) shows that Grh binding is not restricted to chromosome 2.

Development • Supplementary information

Nevil Figure S5

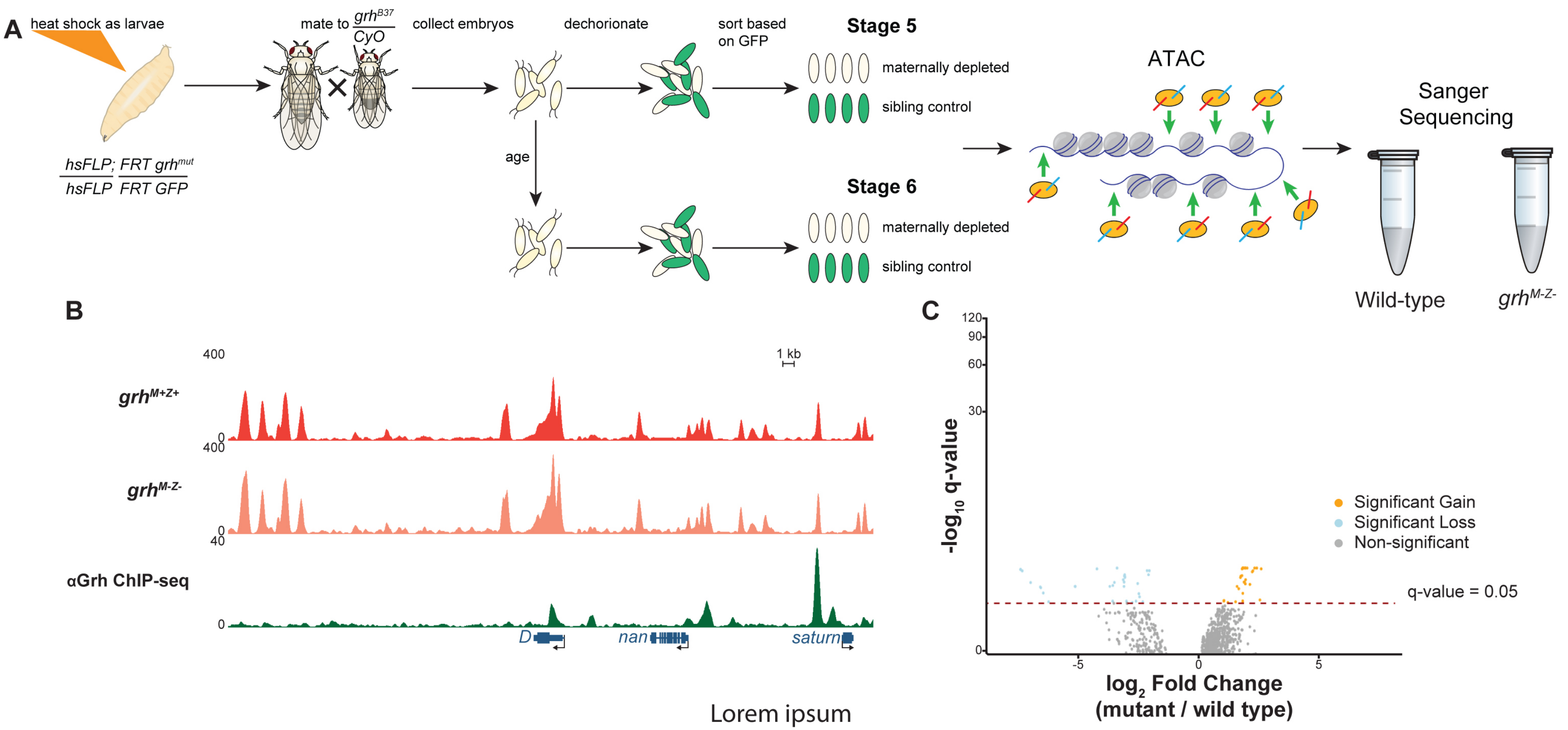

Figure S5. Grh is not required for chromatin accessibility in the early embryo. (A) Scheme used to collect and identify *grh*$^{M-Z-}$ embryos. Follwing heat shock to generate germline mitotic clones lacking maternally encoded Grh, single GFP- and GFP+ embryos were collected for ATAC. After library preparation, the region spanning the *grh*^{B37} deletion was amplified by PCR and sequenced to identify homozygous mutants. (B) UCSC genome browser tracks of a representative locus showing single stage 5 embryos ATAC-seq data either *grh* maternal and zygotic depletions (*grh*$^{M-Z-}$) or wild-type sibling control (*grh*$^{M+Z+}$) along with ChIP-seq peaks for Grh from stage 5 embryos (Nevil et al., 2017). (C) Volcano plot showing all accessible regions identified in the *grh* maternal and zygotic depletion at stage 5. Significance of change in accessibility reported by $-\log_{10}$(q-value) on the y-axis, and magnitude of change by $\log_2$(fold change) on the x-axis. Regions that significantly gain (orange) or lose (blue) accessibility are defined as those with a q-value < 0.05. Non-significant changes are those with a q-value > 0.05 (gray).

Nevil Figure S6

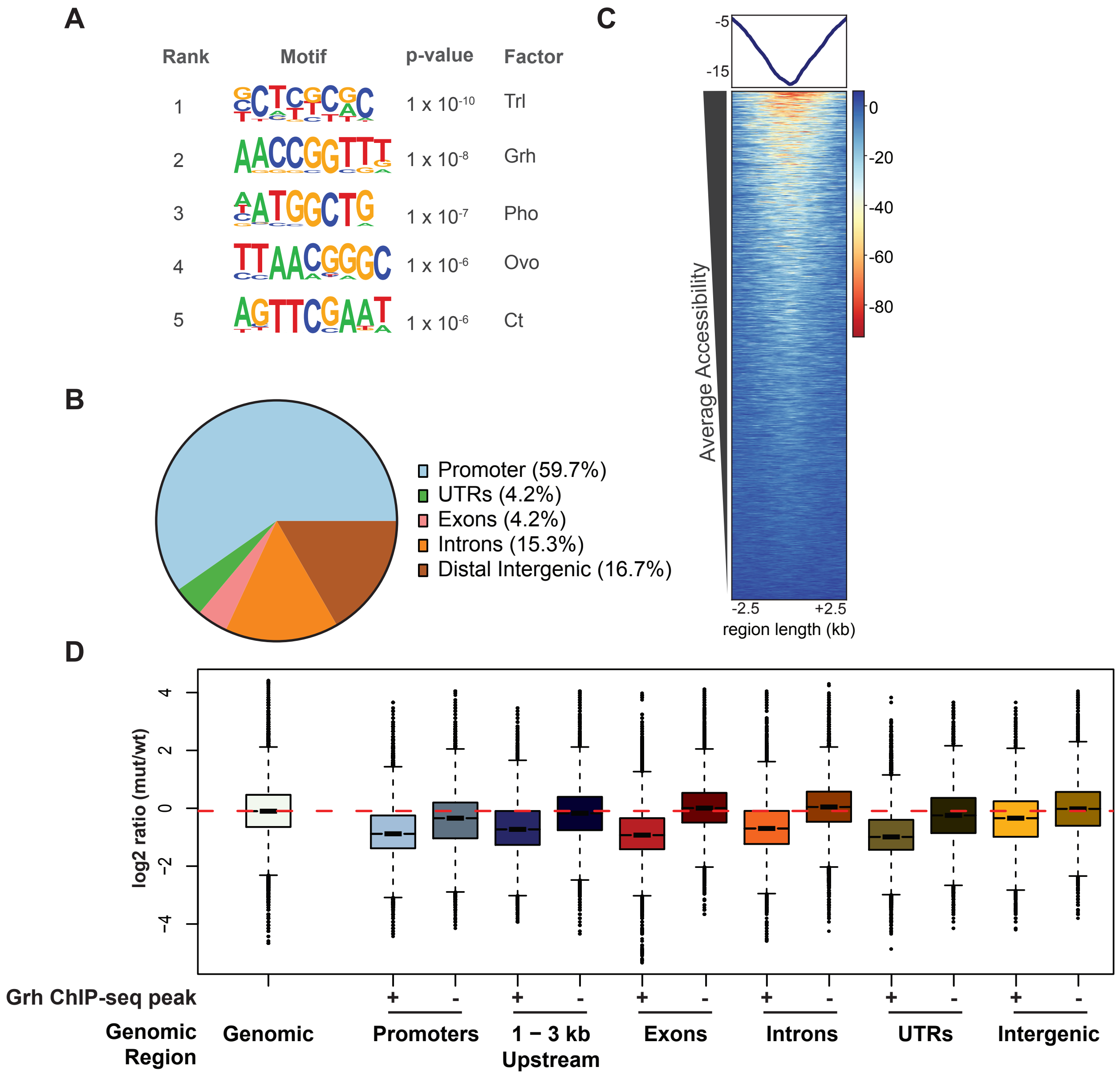

Figure S6. Genomic regions that overlap with a Grh peak lose chromatin accessibility in the absence of Grh. (A) Top 5 motifs identified in *de novo* motif enrichment of regions that change in chromatin accessibility 11-12 hr AEL in *grh*-null mutant embryos (*grh*$^{B37/B37}$). (B) Genomic distribution of regions that change in accessibility and overlap with known Grh ChIP-seq peaks (Nevil et al., 2017). (C) Heatmap of ATAC-seq data from *grh*-mutant and wild-type control embryos for all Grh-bound regions as identified by ChIP-seq. Color scale indicates the difference in relative height of ATAC-seq signal, i.e. accessibility. Data are centered on Grh ChIP-seq peak summits. (D) Changes in chromatin accessibility were calculated by taking random 100 bp windows within the indicated genomic features that either contained a Grh-binding site or did not (Nevil et al., 2017). The $\log_2$ ratio (*grh* mutant/ wild-type) of the read depth normalized for depth and breadth was calculated for each. The "Genomic" group indicates the signal from random genomic regions which are largely free of Grh binding.

Nevil Figure S7

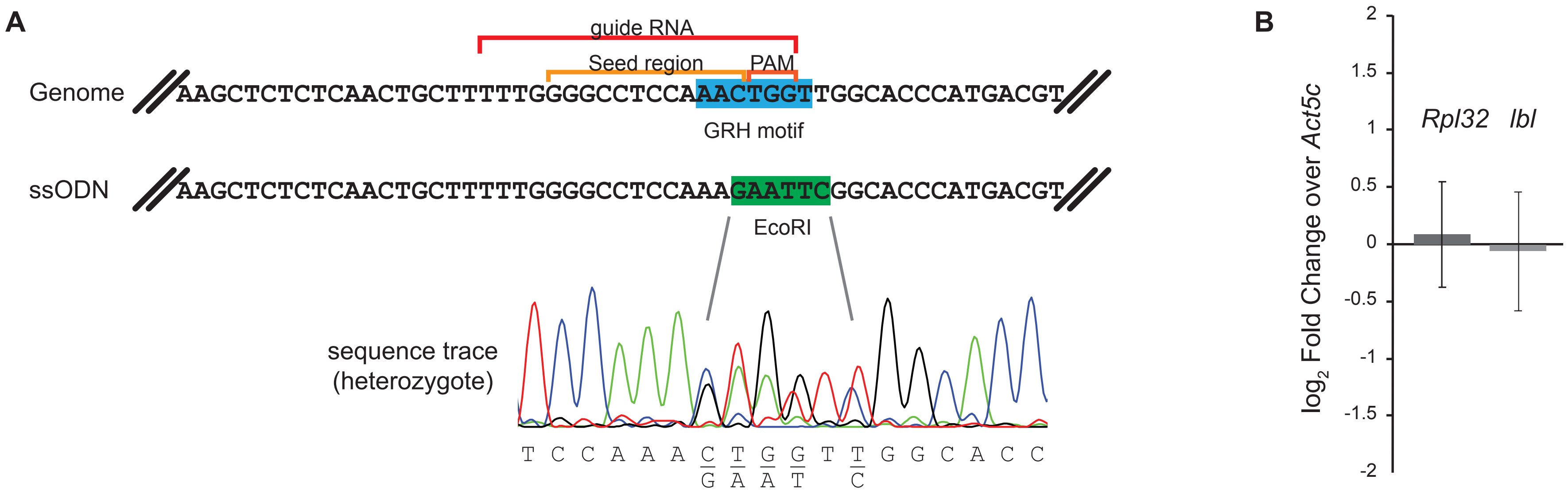

Figure S7. ssODN design and mutation of Grh-binding site in the *Ibl* promoter. (A) A 135-nucleotide single-stranded oligodeoxynucleotide (ssODN) donor template was designed across the *Ibl* promoter to mutate the canonical Grh motif. In the wild-type genomic locus the Grh motif overlaps the guideRNA-recognition site. Successful mutation to an EcoRI site sequence abrogates guideRNA recognition by mutating both the seed region and PAM. Mutants lines were identified and sequenced. (B) $\log_2$ fold change of control *Rpl32* mRNA and *Ibl* mRNA in Ibl^{GBE-} and w^{1118} control 11-12 hr AEL embryos. Data were normalized to an *Act5c* mRNA control.

Development • Supplementary information

Nevil Figure S8

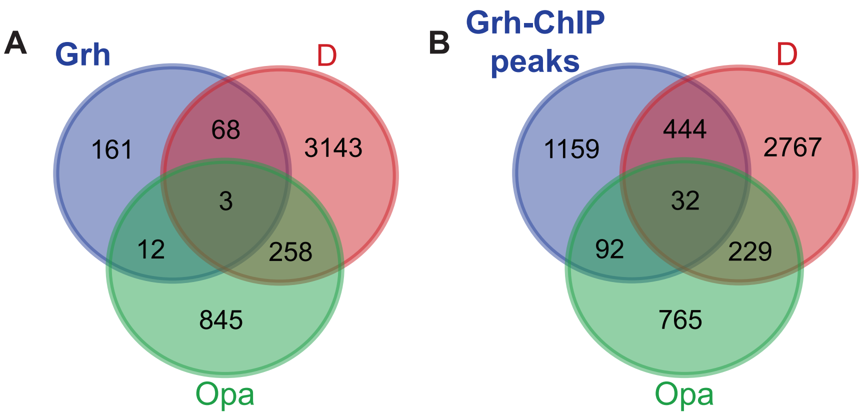

Figure S8. Overlap of Grh, Opa and D binding motifs in regions that increase in chromatin accessibility during gastrulation. (A) Overlap of regions that gain accessibility during gastrulation and contain motifs (as defined by HOMER) for the indicated factors. (B) Overlap of regions that gain accessibility during gastrulation and have a Grh-binding site as defined by ChIP-seq and contain motifs (as defined by HOMER) for Opa or D.

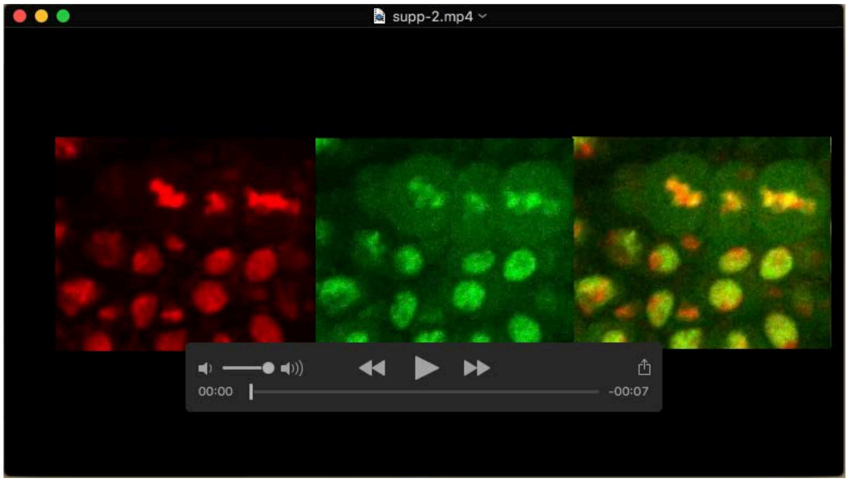

Movie 1. sfGFP-Grh remains bound to chromatin during mitosis during *Drosophila melanogaster* gastrulation.

Time lapse movie of mitotic and interphase cells during gastrulation in a *Drosophila melanogaster* embryo expressing His2Av-RFP (red, left panel) and sfGFP-Grh (green, middle panel). Merged data are presented in the right panel. 49 images were captured 7 seconds apart. Playback rate is 7 frames/second.
