# Supplementary material for: Establishment of chromatin accessibility by the conserved transcription factor Grainy head is developmentally regulated
Source: Development. 2020 Mar 11;147(5):dev185009. doi: 10.1242/dev.185009 (PMC10624965; doi:10.1242/dev.185009)
Supplement: Supplementary file 2 [file develop-147-185009_review_history.pdf]

Establishment of chromatin accessibility by the conserved transcription factor Grainy head is developmentally regulated

Markus Nevil, Tyler J. Gibson, Constantine Bartolutti, Anusha Iyengar and Melissa M. Harrison

DOI: 10.1242/dev.185009

Editor: Susan Strome

Review timeline

Original submission:	25 September 2019
Editorial decision:	11 November 2019
First revision received:	13 January 2020
Editorial decision:	3 February 2020
Second revision received:	3 February 2020
Accepted:	7 February 2020

Original submission

First decision letter

MS ID#: DEVELOP/2019/185009

MS TITLE: Establishment of chromatin accessibility by the conserved transcription factor Grainy head is developmentally regulated

AUTHORS: Markus Nevil, Tyler J. Gibson, Constantine Bartoluttii, Anusha Iyengar, and Melissa M. Harrison

I have now received reviews of your manuscript from 3 experts. The reviewers' comments are appended below, or you can access them online: please go to BenchPress and click on the 'Manuscripts with Decisions' queue in the Author Area.

As you will see, all 3 reviewers express interest in your work, but have some significant concerns, most notably the issue of whether maternal Grh can rescue zygotic mutants and vice versa and whether this study represents a significant advance in understanding developmental gene regulation given that it is already well-appreciated that pioneer transcription factors are subject to context-specific control. All 3 reviewers offer numerous excellent suggestions for revising and improving your manuscript. I invite you to consider the reviewers' suggestions and submit a revised manuscript. Your revised manuscript will be re-reviewed, and acceptance will depend on your satisfactorily addressing the reviewers' concerns and making clear the advance that your manuscript presents. Please note that Development normally permits only one round of 'major revision'.

In your revised manuscript, please clearly HIGHLIGHT all changes made in the revised version. You should avoid using 'Tracked Changes' in Word files as these are lost in PDF conversion. I also request a point-by-point response detailing how you have dealt with the points raised by the reviewers in the 'Response to Reviewers' box. If you do not agree with any of the reviewers' criticisms or suggestions, please explain why.

© 2020. Published by The Company of Biologists under the terms of the Creative Commons Attribution License (https://creativecommons.org/licenses/by/4.0/).

<u>Reviewer 1</u>

Advance summary and potential significance to field

This is a well written, interesting paper. The data clearly show that (1) shGFP-Grh localizes on mitotic chromosomes (2) maternal Grh is not required for chromatin accessibility in early embryos (3) zygotic Grh is not required for chromatin accessibility in early embryos, (4) Grh is required for chromatin accessibility in late embryos, and (5) mutation of a Grh binding site in the lbl locus does not significantly alter chromatin accessibility, suggesting that other factors can take over for Grh at this site. Altogether the results suggest that the “pioneer” ability of Grh, ie, the ability to open chromatin, is stage specific, and can be redundant with other factors. These are important results as they suggest that “pioneer” is not an intrinsic property of the Grh transcription factor (and presumably other transcription factors) but can be a regulated activity.
These results provide new information about the transcriptional control of development.

Comments for the author

I have the following questions/comments:

(1) Regarding Fig. 1. This is quite beautiful. The movie too. It appears that the nucleus in the movie is the same as the one in the Figure. How many nuclei looked like this?
(2) Fig. 2, grhM-, what is the zygotic genotype of these embryos? Could the zygotic component be rescuing the lack of zygotic Grh?
(3) Figure 4, Could maternal component be rescuing the zygotic phenotype? Could the experiment be repeated in maternal, zygotic mutant embryos? If this has not been done it seems like it should be, otherwise I'm not sure you can make the conclusion that Grh is not required for open chromatin at stages 5 and 6. Or please state the evidence that maternal Grh is gone by stage 5/6 or that zygotic Grh is not transcribed/translated at or before stage 5/6.
(4) Supplemental Fig. 1. It’s interesting that some nuclei (yolk nuclei?) are green, ie they have no His2AvRFP why is this?

<u>Reviewer 2</u>

Advance summary and potential significance to field

Review of: “Establishment of chromatin accessibility by the conserved transcription factor Grainy head is developmentally regulated.”
Summary: The focus of this paper is to study the role of the Grh transcription factor in binding and opening genomic DNA. Previous work from the Harrison lab established that Grh binds many of the same genomic locations over developmental time, and the work of others showed that Grh binding can promote chromatin opening in imaginal disc tissues. In this paper, the authors address two further questions in regards to Grh function.
First, they used a genomic targeting strategy to tag the endogenous Grh loci with a super-folder-GFP (sfGFP). They establish that the sfGFP-Grh allele is viable and shows no obvious phenotypes, consistent with the tag not significantly compromising Grh function. The authors then perform live imaging of embryos that express the sfGFP-Grh protein and found that it remains bound to mitotic chromosomes during cell division – a property often found in pioneer transcription factors. This data is clear and convincing.
Second, the authors use ATAC-seq to analyze the genomic opening of wild type Stage 5 and 6 embryos and found thousands of different accessible genomic regions. Bioinformatics analysis focused on those gained at Stage 6 revealed significant motif enrichment for Grh and Forkhead-like (Fox) and Dichaete (Sox), the latter two TFs have pioneer-like activities in mammals. To assess if Grh is required for the formation of these newly accessible regions, the authors generated either maternal Grh null or zygotic Grh null embryos and performed ATAC-seq on similarly aged embryos. Overall, the authors found no significant changes in genomic accessibility in Stage 5 or Stage 6 embryos that lack either maternal or zygotic Grh – suggesting that Grh is not a pioneer TF in the early embryo. Analysis of older embryos in Grh zygotic nulls, however, revealed significant changes in chromatin accessibility. Based on these results the authors argue that Grh is a developmentally regulate pioneer TF.

© 2020. Published by The Company of Biologists under the terms of the Creative Commons Attribution License (https://creativecommons.org/licenses/by/4.0/).

Overall, the experiments performed are done well and the data is presented in a clear manner. However, I do have a major concern as to whether the authors have clearly shown that Grh does not function as a pioneer TF in the early embryo. Without addressing this concern, there are alternative interpretations to the data in regards to the different developmental stages by which Grh functions as a potential pioneer TF.

Comments for the author

Major Concern: The main conclusion of the paper is based on the assumption that it is either the maternal OR zygotic Grh that functions at Stage 6 of embryogenesis – and hence the fact that no significant change in accessibility is observed when one or the other is removed forms the basis of the authors argument that Grh does not function as a pioneer TF at this stage of embryogenesis. But couldn't it simply reflect that there is sufficient maternal Grh or zygotic Grh expressed at Stage 6 to perform this pioneer activity and that one would need to genetically remove both maternal and zygotic Grh to see any defect? Considering that the timing between Stage 5 (130-180min) and Stage 6 (180-195) is tight, isn't it possible that there is sufficient maternal Grh loaded in the zygotic Grh null to trigger accessibility changes at Stage 6 of Grh zygotic nulls but not later in embryogenesis (11-12hour embryos)? Alternatively, isn't it possible that there is sufficient zygotic Grh expressed by Stage 6 to trigger the changes in accessibility in maternal Grh null embryos? Hence, there are two interpretations to the data presented in the paper: The authors interpretation that Grh does not function as a pioneer TF early in the embryo; or an alternative interpretation that there is sufficient Grh expressed in both the maternal and zygotic nulls to fulfill Grh function at stage 6, but there is not sufficient maternal Grh to fulfill Grh function in older embryos (11-12hrs). If the latter is true, it would significantly compromise the main conclusion of the study.

In Figure 6B, the authors assess the ability of Grh to activate the ladybird enhancer/promoter in Drosophila S2 cells. I have two comments on this assay: #1) The authors do not show what the luciferase activity of the wild type and mutant Ladybird-luciferase vectors are in the absence of Grh. Are both the wild type and grh-binding site mutant reporters expressed at similar levels in the absence of Grh or do they show differences in activity in the absence of Grh transfection? Does adding Grh fail to stimulate the Grh binding mutant luciferase? Such experiments would help to better define the direct impact Grh has on the ladybird regulatory element. #2) While this data looks very strong and convincing, there are no statistical tests noted on Figure 6B.

In Figure 6 – the authors argue that Grh is not required for local accessibility at the ladybird late promoter. I have a concern in regards to whether the data supports that claim. In these studies, the authors are using two types of experimental tests to support their conclusions: First, the authors use genomic point mutations to disrupt a Grh binding site in the promoter and they show quite convincingly that Grh binding is lost in embryos in Fig6C. They also measure accessibility of this region using FAIRE-qPCR to show the accessibility is not significantly compromised in wing discs (why they switched tissues in these assays is not clear).
However, the authors also show in Fig 6D, what appears to be a large change in accessibility of the Ladybird promoter in the late embryo Grh mutants as compared to the Grh heterozygous controls. Doesn't this show that Grh function is required for proper ladybird enhancer accessibility in the embryo? Do the authors know if Grh mutant wing discs show a similar loss of ladybird accessibility? If they do see a difference, wouldn't that refute the figure legend title? In general, the authors should be more clear in their text and presentation of the data in terms of changes due to genetic loss of Grh (can have both direct and indirect impacts) versus changes due to the loss of binding site (only a direct effect) and the authors should be more clear when they are comparing data in embryos with data in embryos versus data in embryos with data in wing discs.

<u>Reviewer 3</u>

Advance summary and potential significance to field

In this manuscript, the authors explore the potential gene regulatory roles of the transcription factor Grh in Drosophila. Prior work demonstrated the requirement of Grh for proper chromatin accessibility profiles at a later stage of fly development (larval eye disc), suggesting a potential

© 2020. Published by The Company of Biologists under the terms of the Creative Commons Attribution License (https://creativecommons.org/licenses/by/4.0/).

role of Grh as a pioneer factor. Here, the authors examine whether Grh exhibits behaviors consistent with pioneering activity in early embryos. Using a combination of live imaging, genomics of wild-type and Grh mutant embryos, and CRISPR-mediated mutagenesis of a Grh binding site, the authors find that Grh has a limited role in controlling chromatin accessibility in embryos, leading them to conclude that Grh's pioneering activity is influenced by additional factors. Overall, the manuscript is well-written, the experiments are executed appropriately, and the data are of high quality. The primary consideration is whether this conclusion represents a significant advance to the field of developmental gene regulation. It is already understood that "pioneer" transcription factors are subject to context-specific regulation. Thus, further experimental insight as to why Grh has a role in controlling chromatin accessibility in larval tissues but not in early embryos is necessary. Alternatively, the authors could further explore the role of Grh in gene regulation in early embryos despite not controlling chromatin accessibility at this stage. Prior work from this group already documented changes in the transcriptome of Grh M- or Grh Z- embryos (Nevil et al, 2017); therefore, it might be valuable to examine the relationships between changes in gene expression, chromatin accessibility, and enhancer activity.

Comments for the author

Main comments and suggestions.
• Figure 1/sfGFP-Grh
o Figure 1. Additional controls/comparisons would help interpretation of the Grh signal. For example, what does nuclear-localized sfGFP look like in these experiments? Can the authors compare the Grh signal to their existing zld-GFP signal (which I think does not bind mitotic chromosomes)?
o Line 107: Does the first detectable expression of sfGFP-Grh at gastrulation represent zygotic expression? Can the authors clarify whether these embryos would express maternally-contributed sfGFP-Grh (and if so, why it is not detected)?
• Figure 2.
o Here and throughout the manuscript, it would be useful to know if the loci depicted in the browser shots correspond to Grh binding sites. Can the existing Grh ChIP-seq data be added?
• Figure 3.
o Motif analysis: I would be curious to know if these motifs would also be enriched when all open chromatin sites were used as background instead of the reciprocal accessibility category (as was presented in the paper).
• Grh loss of function at stage 5 and stage 6.
o It would be helpful to have a description of the phenotypes of Grh M- and Grh Z- (e.g. developmental phenotype; gene expression phenotype) at stages 5 and 6 when no defects in chromatin accessibility were observed. In other words, is there a role for Grh independent of controlling chromatin accessibility at these stages of embryogenesis? For this question, it may be valuable to examine the activity of individual Grh target enhancers in transgenic reporter assays.
o Also, please comment on the extent to which Grh maternal contribution could compensate for loss of zygotic Grh expression (and vice versa, can zygotic Grh expression compensate for loss of maternal Grh expression).
o Is the defect on chromatin accessibility simply subtle? For example, is there a decrease in the average ATAC signal at Grh peaks in WT vs. Grh loss of function?
• Figure 4B. It may be worthwhile to highlight ATAC peaks located on chr2 in the volcano plot. The restriction of loss of accessible chromatin to the mutant Grh chromosome is important, but that result is not conveyed in this figure. Looking at the figure without reading the text makes the title of this figure incongruous with the plotted data.
• Figure 5.
o It would be preferred to have the color bars in B and C located elsewhere. In their current location, they look like y-axis/row labels.
o How may Grh ChIP-seq peaks are there in panel C? It seems that all of them are affected by Grh Z-, making it surprising that only 92 sites are identified as differentially accessible. Additional discussion could help explain. Also, it could help to plot a heatmap of the ATAC signal in WT subtracted by the ATAC signal in Grh Z-. This could even be done at other stages too.
o Panel D. The decrease in ATAC signal observed may be even more apparent if the summit of the Grh ChIP-seq signal were used instead of the peak center.
• Loss of accessibility in Grh Z- stage 14/15 embryos.

© 2020. Published by The Company of Biologists under the terms of the Creative Commons Attribution License (https://creativecommons.org/licenses/by/4.0/).

o An expanded description of the 92 sites affected by loss of Grh would be beneficial. E.g. genomic distribution (promoters, enhancers, chromatin colors…). What fraction of these 92 sites are Grh ChIP-seq peaks? Are these 92 sites near to genes regulated by Grh? Is the transcriptional activity of any of these sites affected in transgenic reporter assays?

• Figure 6.

o Panel A. If this site gains accessibility over time in wild-type embryos, as stated in the text, it would help to include these data in the figure.

o Panel E. Why use wing discs for this experiment? Why not use Grh Z- embryos?

o Along similar lines, I am puzzled why the authors did not examine the effects of Grh binding site mutation on lbl gene expression in embryos (or discs).

Minor comments:

• Line 10: it is not sufficiently clear that the work described corresponds to new experiments, or those previously published.
• Line 10: “larvae” should be singular.
• Line 103: “is” missing.
• Line 111: “with” seems to be present in error.

First revision

Author response to reviewers' comments

We appreciate that reviewers generally found the manuscript “well written” and “interesting”, and the “data clear and convincing.” We thank them for their helpful comments. We made changes to the manuscript (detailed below and highlighted) to address all the concerns and believe the manuscript is significantly strengthened.

All reviewers wanted us to determine whether maternally expressed Grh could persist and compensate for the lack of zygotic Grh at stage 6, and/or that some zygotic Grh is expressed prior to gastrulation and compensates for the lack of maternal Grh at stage 5.

To address these concerns, we generated embryos lacking both maternally contributed and zygotically expressed Grh and tested for changes in chromatin accessibility at both stage 5 and stage 6. The results for these experiments are presented in Figure 4C,D and a newly generated Figure S5. These data demonstrate that loss of both potential sources of Grh does not result in any significant change in chromatin accessibility and are discussed on lines 207-214. Thus, Grh is not required to determine the chromatin accessibility landscape in the early embryo.

Below we have detailed responses to each of the reviewers’ comments.

Reviewer 1

This is a well written, interesting paper. The data clearly show that (1) shGFP-Grh localizes on mitotic chromosomes (2) maternal Grh is not required for chromatin accessibility in early embryos (3) zygotic Grh is not required for chromatin accessibility in early embryos, (4) Grh is required for chromatin accessibility in late embryos, and (5) mutation of a Grh binding site in the lbl locus does not significantly alter chromatin accessibility, suggesting that other factors can take over for Grh at this site. Altogether the results suggest that the “pioneer” ability of Grh, ie, the ability to open chromatin, is stage specific, and can be redundant with other factors. These are important results as they suggest that “pioneer” is not an intrinsic property of the Grh transcription factor (and presumably other transcription factors) but can be a regulated activity. These results provide new information about the transcriptional control of development.

(1) Regarding Fig. 1. This is quite beautiful. The movie too. It appears that the nucleus in the movie is the same as the one in the Figure. How many nuclei looked like this?

© 2020. Published by The Company of Biologists under the terms of the Creative Commons Attribution License (https://creativecommons.org/licenses/by/4.0/).

We thank the reviewer for appreciating the beauty of these data. While we did not quantify the number of nuclei that showed this striking mitotic retention, all nuclei with sfGFP-GRH signal that were captured or observed during mitosis exhibited chromatin binding. We have included additional examples of mitotic chromatin binding in Figure S1B and S1C to further illustrate this point. We have also revised the figure legend to specify that the nuclei in Figure 1 are the same nuclei presented in the movie.

(2) Fig. 2, grhM-, what is the zygotic genotype of these embryos? Could the zygotic component be rescuing the lack of zygotic Grh?

See discussion above in response to this concern raised by all three reviewers.

(3) Figure 4, Could maternal component be rescuing the zygotic phenotype? Could the experiment be repeated in maternal, zygotic mutant embryos? If this has not been done it seems like it should be, otherwise I'm not sure you can make the conclusion that Grh is not required for open chromatin at stages 5 and 6. Or please state the evidence that maternal Grh is gone by stage 5/6 or that zygotic Grh is not transcribed/translated at or before stage 5/6.

See discussion above in response to this concern raised by all three reviewers.

(4) Supplemental Fig. 1. It's interesting that some nuclei (yolk nuclei?) are green, ie they have no His2AvRFP, why is this?

We do not believe that these structures are yolk nuclei, but instead are autofluorescence that we have seen in other backgrounds. The literature suggests that these autofluorescent granules are yolk protein (Andersen and Horne-Badovinac, Development 2016). As confirmation, we imaged w^{1118} embryos at the same stage and observed the same autofluorescence without any fluorescent markers present (see attached image).

Gastrulating w^{1118} embryo (approx. stage 6)

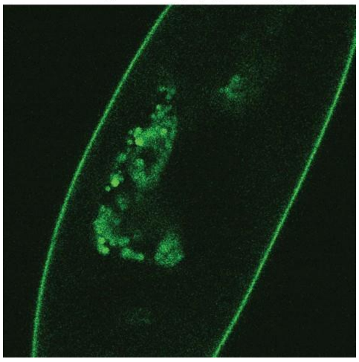

Reviewer 2

Summary: The focus of this paper is to study the role of the Grh transcription factor in binding and opening genomic DNA. Previous work from the Harrison lab established that Grh binds many of the same genomic locations over developmental time, and the work of others showed that Grh binding can promote chromatin opening in imaginal disc tissues. In this paper, the authors address two further questions in regards to Grh function.

First, they used a genomic targeting strategy to tag the endogenous Grh loci with a super-folder-GFP (sfGFP). They establish that the sfGFP-Grh allele is viable and shows no obvious phenotypes, consistent with the tag not significantly compromising Grh function. The authors then perform live imaging of embryos that express the sfGFP-Grh protein and found that it remains bound to mitotic chromosomes during cell division – a property often found in pioneer transcription factors. This data is clear and convincing.

Second, the authors use ATAC-seq to analyze the genomic opening of wild type Stage 5 and 6 embryos and found thousands of different accessible genomic regions. Bioinformatics analysis focused on those gained at Stage 6 revealed significant motif enrichment for Grh and Forkhead-like (Fox) and Dichaete (Sox), the latter two TFs have pioneer-like activities in mammals. To assess if Grh is required for the formation of these newly accessible regions, the authors generated either maternal Grh null or zygotic Grh null embryos and performed ATAC-seq on similarly aged embryos. Overall, the authors found no significant changes in genomic accessibility in Stage 5 or Stage 6 embryos that lack either maternal or zygotic Grh – suggesting that Grh is not a pioneer TF in the early embryo. Analysis of older embryos in Grh zygotic nulls, however,

© 2020. Published by The Company of Biologists under the terms of the Creative Commons Attribution License (https://creativecommons.org/licenses/by/4.0/).

revealed significant changes in chromatin accessibility. Based on these results the authors argue that Grh is a developmentally regulate pioneer TF.

Overall, the experiments performed are done well and the data is presented in a clear manner. However, I do have a major concern as to whether the authors have clearly shown that Grh does not function as a pioneer TF in the early embryo. Without addressing this concern, there are alternative interpretations to the data in regards to the different developmental stages by which Grh functions as a potential pioneer TF.

Major Concern: The main conclusion of the paper is based on the assumption that it is either the maternal OR zygotic Grh that functions at Stage 6 of embryogenesis – and hence the fact that no significant change in accessibility is observed when one or the other is removed forms the basis of the authors argument that Grh does not function as a pioneer TF at this stage of embryogenesis. But couldn't it simply reflect that there is sufficient maternal Grh or zygotic Grh expressed at Stage 6 to perform this pioneer activity and that one would need to genetically remove both maternal and zygotic Grh to see any defect? Considering that the timing between Stage 5 (130-180min) and Stage 6 (180-195) is tight, isn't it possible that there is sufficient maternal Grh loaded in the zygotic Grh null to trigger accessibility changes at Stage 6 of Grh zygotic nulls, but not later in embryogenesis (11-12hour embryos)? Alternatively, isn't it possible that there is sufficient zygotic Grh expressed by Stage 6 to trigger the changes in accessibility in maternal Grh null embryos? Hence, there are two interpretations to the data presented in the paper: The authors interpretation that Grh does not function as a pioneer TF early in the embryo; or an alternative interpretation that there is sufficient Grh expressed in both the maternal and zygotic nulls to fulfill Grh function at stage 6, but there is not sufficient maternal Grh to fulfill Grh function in older embryos (11-12hrs). If the latter is true, it would significantly compromise the main conclusion of the study.

See the discussion above in response to this concern raised by all three reviewers.

(2) In Figure 6B, the authors assess the ability of Grh to activate the ladybird enhancer/promoter in Drosophila S2 cells. I have two comments on this assay: #1) The authors do not show what the luciferase activity of the wild type and mutant Ladybird-luciferase vectors are in the absence of Grh. Are both the wild type and grh-binding site mutant reporters expressed at similar levels in the absence of Grh or do they show differences in activity in the absence of Grh transfection? Does adding Grh fail to stimulate the Grh binding mutant luciferase? Such experiments would help to better define the direct impact Grh has on the ladybird regulatory element. #2) While this data looks very strong and convincing, there are no statistical tests noted on Figure 6B.

We are glad that the reviewer finds these data strong and convincing. Our dual-luciferase assays are performed with and without plasmids allowing expression of Grh. This allows us to normalize our data to account for differences in expression of the reporters and thus to report only the effects of Grh-mediated activation on these reporters. The data presented are shown as the fold activation induced by Grh expression as compared to reporter expression in the absence of Grh. We discuss this in the Methods (lines 502-503). We have also included the overlooked statistical test that is now presented in the figure and figure legend.

(3) In Figure 6 – the authors argue that Grh is not required for local accessibility at the ladybird late promoter. I have a concern in regards to whether the data supports that claim. In these studies, the authors are using two types of experimental tests to support their conclusions: First, the authors use genomic point mutations to disrupt a Grh binding site in the promoter and they show quite convincingly that Grh binding is lost in embryos in Fig6C. They also measure accessibility of this region using FAIRE-qPCR to show the accessibility is not significantly compromised in wing discs (why they switched tissues in these assays is not clear). However, the authors also show in Fig 6D, what appears to be a large change in accessibility of the Ladybird promoter in the late embryo Grh mutants as compared to the Grh heterozygous controls. Doesn't this show that Grh function is required for proper ladybird enhancer accessibility in the embryo? Do the authors know if Grh mutant wing discs show a similar loss of ladybird accessibility? If they do see a difference, wouldn't that refute the figure legend title? In general, the authors should be more clear in their text and presentation of the data in terms of changes due to genetic loss of

© 2020. Published by The Company of Biologists under the terms of the Creative Commons Attribution License (https://creativecommons.org/licenses/by/4.0/).

Grh (can have both direct and indirect impacts) versus changes due to the loss of binding site (only a direct effect) and the authors should be more clear when they are comparing data in embryos with data in embryos versus data in embryos with data in wing discs.

We thank the reviewers for bringing these concerns to our attention. We apologize for the lack of clarity in the text and have revised the results to better explain our experimental design (lines 268-275).

As mentioned by the reviewer, we identified a significant change in accessibility at the *lbl* promoter in the *grh* mutant embryos, but wanted to begin to address whether this change was directly due to Grh binding or indirectly due to Grh effects on gene expression (as mentioned by the reviewer), For this purpose, we mutated the Grh-binding site. However, in our FAIRE experiments we sought to avoid issues with cell type heterogeneity that would adversely affect our ability to detect the full extent to changes in chromatin accessibility in the embryo and might mask effects of mutating the Grh-binding site. Because the *lbl* promoter is both bound by Grh and accessible in the wing disc and the 11-12 hour embryo (Figure 6A, C), we chose to assay accessibility in the wing disc where all of the tissue expresses Grh (Figure S1). (Because *grh* mutants die as embryos, it is not straightforward to assess chromatin accessibility in larval mutant wing discs.)

To address the question of whether *lbl* expression changes, we have included additional data where we examined *lbl* gene expression in the 11-12 hr AEL embryo (Figure S7B) indicating that there is no change in expression of *lbl* when the Grh motif is lost, further supporting the model that accessibility is maintained when Grh binding is abrogated at the promoter. We recognize the limitations of the conclusions that can be drawn from these imperfect experiments, but felt it important (as mentioned by the reviewer) to begin to address the question of direct vs. indirect effects within an endogenous context. We have worked to modify the text to reflect these complicating issues.

Reviewer 3

In this manuscript, the authors explore the potential gene regulatory roles of the transcription factor Grh in Drosophila. Prior work demonstrated the requirement of Grh for proper chromatin accessibility profiles at a later stage of fly development (larval eye disc), suggesting a potential role of Grh as a pioneer factor. Here, the authors examine whether Grh exhibits behaviors consistent with pioneering activity in early embryos. Using a combination of live imaging, genomics of wild-type and Grh mutant embryos, and CRISPR-mediated mutagenesis of a Grh binding site, the authors find that Grh has a limited role in controlling chromatin accessibility in embryos, leading them to conclude that Grh's pioneering activity is influenced by additional factors. Overall, the manuscript is well-written, the experiments are executed appropriately, and the data are of high quality. The primary consideration is whether this conclusion represents a significant advance to the field of developmental gene regulation. It is already understood that "pioneer" transcription factors are subject to context-specific regulation. Thus, further experimental insight as to why Grh has a role in controlling chromatin accessibility in larval tissues but not in early embryos is necessary. Alternatively, the authors could further explore the role of Grh in gene regulation in early embryos despite not controlling chromatin accessibility at this stage. Prior work from this group already documented changes in the transcriptome of Grh M- or Grh Z- embryos (Nevil et al, 2017); therefore, it might be valuable to examine the relationships between changes in gene expression, chromatin accessibility, and enhancer activity.

We thank the reviewer for their positive assessment of the writing, experiments and high-quality of the data. We agree that previous work has shown that a subset of pioneer factors are subject to context-specific regulation. However, nearly all of these studies have been performed in culture. Few studies have focused on pioneer factors within the context of a developing organism. We believe this current submission therefore significantly expands our understanding of how pioneer factors function in development. In addition, our data separate mitotic retention from pioneering activity. Together, this work represents a significant advance and provides the foundation for additional mechanistic studies.

© 2020. Published by The Company of Biologists under the terms of the Creative Commons Attribution License (https://creativecommons.org/licenses/by/4.0/).

We appreciate the importance of understanding why Grh function in accessibility changes over development, and this is an ongoing area of research in the lab. However, this is a non-trivial experimental question that we believe goes beyond the current scope of this manuscript. To begin to identify factors that may regulate accessibility in the embryo, we performed motif searches (lines 164-181). Indeed, newly published data from the Blythe and Stathapolous labs beautifully demonstrate a role for Opa, whose motif we identified, in driving changes in accessibility in the early embryo. These newly published data support our motif analysis and suggest co-expression of Opa might be one element that determines the necessity for Grh in determining chromatin accessibility. We also agree that it is valuable to examine the relationship between changes in gene expression, chromatin accessibility, and enhancer activity. Our submission has already begun to address this. For this reason, we used genome engineering to interrogate the *lbl* locus to try to tease apart direct from indirect effects. These data suggested that Grh binding was not required at this region for chromatin accessibility and gene expression. Nonetheless, it is clear that the complex relationship between chromatin accessibility, gene expression and enhancer activity, which is made more complicated by the role of both direct and indirect effects, makes it challenging to perform these important gene-editing experiments on a broad scale.

(1) Figure 1/sfGFP-Grh: Figure 1. Additional controls/comparisons would help interpretation of the Grh signal. For example, what does nuclear-localized sfGFP look like in these experiments? Can the authors compare the Grh signal to their existing zld-GFP signal (which I think does not bind mitotic chromosomes)?

Thank you for the suggestion. We have not generated embryos expressing nuclear-localized sfGFP alone, but we have published work demonstrating that the sfGFP-Zld is nuclear during interphase but is not retained on mitotic chromatin (Dufourt, et al., Nat Comm. 2018). This is the same sfGFP used to tag Grh, and thus we believe that the behavior seen in this work is due to Grh, not the tag.

(2) Line 107: Does the first detectable expression of sfGFP-Grh at gastrulation represent zygotic expression? Can the authors clarify whether these embryos would express maternally- contributed sfGFP-Grh (and if so, why it is not detected)?

These embryos are homozygous for sfGFP-Grh and therefore both maternally contributed and zygotically expressed Grh should be tagged. We believe that the expression pattern of sfGFP-Grh reflects increased levels of Grh observed by both RNA (modENCODE) and protein expression (immunoblots) during gastrulation.

(3) Figure 2: Here and throughout the manuscript, it would be useful to know if the loci depicted in the browser shots correspond to Grh binding sites. Can the existing Grh ChIP-seq data be added?

Thank you for this suggestion. Existing, stage-matched Grh ChIP-seq has been added to all genome browser figures.

(4) Figure 3: Motif analysis: I would be curious to know if these motifs would also be enriched when all open chromatin sites were used as background instead of the reciprocal accessibility category (as was presented in the paper).

We have performed this analysis and find that it closely matches the conclusions of our previous motif search. These results have been added in Figure S3C and S3D.

(5) It would be helpful to have a description of the phenotypes of Grh M- and Grh Z- (e.g. developmental phenotype; gene expression phenotype) at stages 5 and 6 when no defects in chromatin accessibility were observed. In other words, is there a role for Grh independent of controlling chromatin accessibility at these stages of embryogenesis? For this question, it may be valuable to examine the activity of individual Grh target enhancers in transgenic reporter assays.

© 2020. Published by The Company of Biologists under the terms of the Creative Commons Attribution License (https://creativecommons.org/licenses/by/4.0/).

At stages 5/6 we have seen no obvious developmental phenotypes for either maternal depletions or zygotic depletions of Grh. Additionally, we are unaware of any literature support for phenotypes beyond modifications to gene expression at this stage. Animals homozygous for null mutants in *grh*, including the B37 allele used here, die late in embryogenesis (Bray and Kafatos Genes Dev 1991). However, we and others have previously shown that there are changes in gene expression when embryos are depleted of either maternal or zygotic Grh (Liaw et al. Genes Dev 1995; Huang et al. Genes Dev 1995; Nevil et al. Genetics, 2017). These data clearly support a non-essential role for Grh at these stages of embryogenesis.

(6) Also, please comment on the extent to which Grh maternal contribution could compensate for loss of zygotic Grh expression (and vice versa, can zygotic Grh expression compensate for loss of maternal Grh expression).

See the discussion above in response to this concern raised by all three reviewers.

(7) Is the defect on chromatin accessibility simply subtle? For example, is there a decrease in the average ATAC signal at Grh peaks in WT vs. Grh loss of function?

We thank the reviewer for their careful thought in examining our data. We believe that even subtle effects would have been captured by our analysis. This is supported by the fact that even in stage 14/15 embryos, where there is significant heterogeneity in cell type, our ATAC-seq data analysis detected loss of chromatin accessibility around Grh-bound regions. Additionally, our analysis is sensitive enough to detect changes in read depth attributable to mutations on the grh^{B37} chromosome.

(8) Figure 4B: It may be worthwhile to highlight ATAC peaks located on chr2 in the volcano plot. The restriction of loss of accessible chromatin to the mutant Grh chromosome is important, but that result is not conveyed in this figure. Looking at the figure without reading the text makes the title of this figure incongruous with the plotted data.

Thank you for the suggestion, we have added this plot to Figure S4C to more clearly show the restriction of the loss of accessible chromatin to chromosome 2.

(9) Figure 5: It would be preferred to have the color bars in B and C located elsewhere. In their current location, they look like y-axis/row labels.

We have modified this figure as requested.

(10) How may Grh ChIP-seq peaks are there in panel C? It seems that all of them are affected by Grh Z-, making it surprising that only 92 sites are identified as differentially accessible. Additional discussion could help explain. Also, it could help to plot a heatmap of the ATAC signal in WT subtracted by the ATAC signal in Grh Z-. This could even be done at other stages too.

Due to the heterogeneity of the cell populations at this stage embryo, we believe that the effects of the loss of Grh are being muted by ATAC signal arising from cells that do not normally express Grh. Thus, by stringent means we only find 92 sites with high confidence. However, as mentioned by the reviewer panel C demonstrates a general loss of accessibility around Grh sites. As you suggest, we have included a heat map of the WT subtracted by the ATAC signal in Grh Z- in Figure S6A.

(11) Panel D. The decrease in ATAC signal observed may be even more apparent if the summit of the Grh ChIP-seq signal were used instead of the peak center.

These data were generated using the summit of the Grh ChIP-seq signal. Thank you for bringing this confusion to our attention. The text of the figure legend has been modified to clarify our analysis.

(12) An expanded description of the 92 sites affected by loss of Grh would be beneficial. E.g. genomic distribution (promoters, enhancers, chromatin colors…). What fraction of these 92 sites

© 2020. Published by The Company of Biologists under the terms of the Creative Commons Attribution License (https://creativecommons.org/licenses/by/4.0/).

are Grh ChIP-seq peaks? Are these 92 sites near to genes regulated by Grh? Is the transcriptional activity of any of these sites affected in transgenic reporter assays?

In Figure S6, we have included a *de novo* motif search and genomic distribution to further characterize the sites affected by the loss of Grh. We also note in the text that 73% of these sites are bound by Grh (lines 226-227) While we did not perform transgenic reporter assays, the *in vivo* mutation of Grh motif in the *lbl* promoter region identified in this analysis did not show any change in gene expression (Figure S7B).

(13) Figure 6 Panel A. If this site gains accessibility over time in wild-type embryos, as stated in the text, it would help to include these data in the figure.

We have modified Figure 6A as requested.

Minor comments:

Line 10: it is not sufficiently clear that the work described corresponds to new experiments, or those previously published.

Line 10: “larvae” should be singular.

Line 103: “is” missing.

Line 111: “with” seems to be present in error.

Thank you for these suggestions, we have made corrections as requested.

<u>Second decision letter</u>

MS ID#: DEVELOP/2019/185009

MS TITLE: Establishment of chromatin accessibility by the conserved transcription factor Grainy head is developmentally regulated

AUTHORS: Markus Nevil, Tyler J Gibson, Constantine Bartolutti, Anusha Iyengar, and Melissa M Harrison

I have now received reviews of your manuscript from the original 3 reviewers. Their comments are appended below, or you can access them online: please go to BenchPress and click on the 'Manuscripts with Decisions' queue in the Author Area.

As you will see, all 3 reviewers are pleased with your revisions and recommend publishing your study in Development. Before I officially accept your manuscript, please consider the suggestion of Reviewer 3 to incorporate into your paper 3 of your responses to reviewers, to provide more context for readers.

Please submit a revised manuscript, highlighting your final changes. You should avoid using 'Tracked Changes' in Word files as these are lost in PDF conversion. Please also provide a point-by-point response detailing how you have dealt with the suggestions of Reviewer 3.

© 2020. Published by The Company of Biologists under the terms of the Creative Commons Attribution License (https://creativecommons.org/licenses/by/4.0/).

Reviewer 1

Advance summary and potential significance to field

In this revised manuscript, the authors have greatly strengthened their paper by performing ATAC-seq on maternal, zygotic grh mutants. This data solidifies their conclusion that Grh is not required for chromatin accessibility in embryos, even though it is retained at mitotic chromosomes at this stage. The authors have now clearly shown that the pioneer activity of the transcription factor Grh is not required for embryogenesis.
Comments for the author

I appreciate the detailed responses to my and the other reviewers' comments and request no additional revisions.

Reviewer 2

Advance summary and potential significance to field

The focus of this paper is to study the role of the Grh transcription factor in binding and opening genomic DNA in the Drosophila embryo. Previous work from the Harrison lab established that Grh binds many of the same genomic locations over developmental time, and the work of others showed that Grh binding can promote chromatin opening in imaginal disc tissues. In this paper, the authors address two further questions in regards to Grh function. First, they used a genomic targeting strategy to tag the endogenous Grh loci with a super-folder-GFP (sfGFP) and show that the sfGFP-Grh protein remains bound to mitotic chromosomes during cell division – a property often found in pioneer transcription factors. Second, the authors used genomic accessibility assays to show that unlike in the imaginal disc tissues, Grh is not required for establishing open chromatin regions in early Drosophila embryos. These data support the model that the pioneer TF activity of Grh is developmentally regulated. The revised paper addressed my major concerns and thus, I believe the paper has made a significant advance and should be published in Development.

Comments for the author

The authors did a great job of addressing all of my comments as well as those of the other two reviewers.
Thus, I am strongly supportive of publication.

Reviewer 3

Advance summary and potential significance to field

This manuscript describes the role played by the transcription factor Grh in transcriptional regulation in Drosophila. Grh exhibits pioneer factor function in later stages of development. Grh is also maternally deposited and zygotically expressed in embryos, raising the question as to whether Grh also functions as a pioneer in embryos. The authors demonstrate that Grh indeed does influence chromatin accessibility, but only in late-stage embryos, despite a documented role in controlling gene expression at this stage (prior study) and binding to mitotic chromatin in early embryos (this study). Thus, this work supports a model in which the ability of factors to control chromatin accessibility is developmentally regulated (as opposed to being an intrinsic property of the transcription factor).

Comments for the author

The authors have satisfactorily addressed my questions and comments in their response letter. My only remaining request is to incorporate several of their responses into the manuscript results or discussion. This will strengthen the manuscript and provide more context for readers to interpret the findings.

© 2020. Published by The Company of Biologists under the terms of the Creative Commons Attribution License (https://creativecommons.org/licenses/by/4.0/).

(1) The manuscript would be strengthened by stating that sfGFP-Zld is not retained on mitotic chromatin (Reviewer 3, point 1 Response).
(2) The authors should state that no obvious developmental phenotypes are observed at stages 5/6 (point 5 Response).
(3) The authors should state cellular heterogeneity likely masks some of the effects on chromatin accessibility in stage 11/12 Grh mutants (point 10 Response).

Second revision

Author response to reviewers' comments

We appreciate the Reviewer's attention to detail and have included the suggested responses. The line numbers for each are provided.

The authors have satisfactorily addressed my questions and comments in their response letter. My only remaining request is to incorporate several of their responses into the manuscript results or discussion. This will strengthen the manuscript and provide more context for readers to interpret the findings.

(1) The manuscript would be strengthened by stating that sfGFP-Zld is not retained on mitotic chromatin (Reviewer 3, point 1 Response).
-This is now included on lines 115-120.

(2) The authors should state that no obvious developmental phenotypes are observed at stages 5/6 (point 5 Response).
-This is now included on lines 147-149.

(3) The authors should state cellular heterogeneity likely masks some of the effects on chromatin accessibility in stage 11/12 Grh mutants (point 10 Response).
-This is now included on lines 223-225.

Third decision letter

MS ID#: DEVELOP/2019/185009

MS TITLE: Establishment of chromatin accessibility by the conserved transcription factor Grainy head is developmentally regulated

AUTHORS: Markus Nevil, Tyler J Gibson, Constantine Bartolutti, Anusha Iyengar, and Melissa M Harrison
ARTICLE TYPE: Research Article

I am happy to tell you that your manuscript has been accepted for publication in Development, pending our standard ethics checks. I did not send it out for re-review but instead assessed your resubmission myself. Thank you for incorporating the reviewer's suggested revisions. Please upload a "clean" copy of your paper with no highlights and no tracked changes to the Development site or email it to dev@biologists.com.

© 2020. Published by The Company of Biologists under the terms of the Creative Commons Attribution License (https://creativecommons.org/licenses/by/4.0/).
